# Abnormal arterial-venous fusions and fate specification in mouse embryos lacking blood flow

**DOI:** 10.1038/s41598-017-12353-z

**Published:** 2017-09-20

**Authors:** Jennifer J. Hwa, Nathan Beckouche, Lawrence Huang, Yoseph Kram, Henrik Lindskog, Rong A. Wang

**Affiliations:** Laboratory for Accelerated Vascular Research, Division of Vascular Surgery, Department of Surgery, University of California, San Francisco, San Francisco, CA 94143 USA

## Abstract

The functions of blood flow in the morphogenesis of mammalian arteries and veins are not well understood. We examined the development of the dorsal aorta (DA) and the cardinal vein (CV) in *Ncx1* ^−/−^ mutants, which lack blood flow due to a deficiency in a sodium calcium ion exchanger expressed specifically in the heart. The mutant DA and CV were abnormally connected. The endothelium of the *Ncx1* ^−/−^ mutant DA lacked normal expression of the arterial markers ephrin-B2 and Connexin-40. Notch1 activation, known to promote arterial specification, was decreased in mutant DA endothelial cells (ECs), which ectopically expressed the venous marker Coup-TFII. These findings suggest that flow has essential functions in the DA by promoting arterial and suppressing venous marker expression. In contrast, flow plays a lesser role in the CV, because expression of arterial-venous markers in CV ECs was not as dramatically affected in *Ncx1* ^−/−^ mutants. We propose a molecular mechanism by which blood flow mediates DA and CV morphogenesis, by regulating arterial-venous specification of DA ECs to ensure proper separation of the developing DA and CV.

## Introduction

The circulatory system is the first organ to emerge during embryonic development because the embryo grows in size and must deliver an adequate blood supply to its tissues. The development of the vascular system is a fundamental process that requires arterial-venous (AV) fate specification in the formation of arteries and veins. In mammalian embryos, the first artery/vein pair to form is the parallel dorsal aorta (DA) and the cardinal vein (CV), which, respectively, carry blood away from and return blood back to the heart. In the mouse embryo, the DA is assembled first from the *de novo* differentiation of angioblast precursors beginning around 3 somite stage (ss), embryonic day (E) 8.0, whereas the CV arises about half a day later^1–3^. The relative simplicity of this first artery/vein pair provides an excellent model for studying artery and vein morphogenesis.

Blood flow plays a critical, yet poorly understood, role in the formation of blood vessels, and in particular the formation of the DA and CV. The heartbeat in the mouse embryo begins at approximately 3ss and blood flow is initiated at around 6–7ss^4^. In mice lacking the giant muscle protein gene *titin*, which is expressed in cardiac and skeletal muscle and required for sarcomere formation and assembly, the embryonic heart displays only weak spontaneous contractions and never develops a regular contractile rhythm, leading to a delayed onset of blood circulation until after E9.5^5,6^. Although *titin* is not expressed in the vasculature, the lumen of the DA in these mutants was variable and irregularly sized at E8.5. This finding suggests that compromised blood flow affects the morphogenesis of the DA and CV. However, the precise function of flow on the development of the DA and CV and the mechanisms underlying its effects remain to be elucidated.

Other mouse flow mutants have also been used to study the requirement of blood flow on mammalian vascular development. For example, embryos with a deficiency in *mlc2a*, a myosin light chain isoform expressed in cardiac muscle, have impaired atrial contractility, and thus, impaired blood flow patterns. These mutants display disorganization in the cranial and intersomitic vessels^4,6,7^. Another widely used model is the *Ncx1* ^−/−^ mutant, which is defective in a sodium calcium ion exchanger expressed specifically in the embryonic heart, and never develops a heartbeat nor blood flow^8–12^. *Ncx1* expression has been shown to be restricted to the heart throughout early embryogenesis until E11.0^11–13^, ensuring that defects seen outside the heart before this stage are a result of the lack of blood flow and not a direct effect of gene function. These mutants have been previously used to demonstrate the essential role of blood flow in hematopoietic stem cell development^8–10,14^. However the development of the DA and CV in *Ncx1* ^−/−^ mutants has not been reported. These mutants provide excellent genetic tools for ascertaining the requirement of blood flow on blood vessel morphogenesis.

Genes in the Notch and the EphB4/ephrin-B2 signaling pathways have been implicated in formation of the mammalian DA and CV^15–18^. Our lab has reported that DA and CV formation may occur in a coordinated manner involving the distribution of endothelial cells (ECs) between the two vessels^19,20^. In addition, mutants in the Notch and the EphB4/ephrin-B2 signaling pathways were found to contain AV shunts, or AV fusions, abnormal, direct connections between the artery and vein that led to a short circuit in circulation^21^. However, how blood flow affects the expression of these genes in the developing DA and CV is not well understood.

Recent studies have begun to shed light on genes regulated by blood flow during artery and vein morphogenesis in the developing mouse embryo. Cultured embryos with physical ablation of blood flow were generated *ex vivo* by snipping the yolk sac vessel inlets to the heart prior to the onset of blood flow to stop blood circulation. These embryos exhibit defects in the vasculature of the extra-embryonic yolk sac, which remains as a primitive capillary-like plexus and fails to remodel into a mature hierarchical artery-vein tree^4,22^. *In situ* hybridization has shown that these cultured embryos lack expression of both *Connexin-40*, an arterial specific marker, as well as *Notch1*, which is expressed arterially to promote the arterial EC fate, in the DA^1,23,24^. However, whether DA and CV morphogenesis is affected by physical ablation of blood flow is unknown.

To investigate the role of blood flow in DA and CV development in utero, we examined their formation in the *Ncx1* ^−/−^ mutant. By imaging DA and CV development in whole mount embryos, we show that in the absence of flow, fusions are found between the DA and CV, suggesting that blood flow plays a crucial role in the proper separation of this artery and vein pair. Fusions were accompanied by defects in arterial-venous specification of the DA, with loss of arterial markers along with ectopic expression of a venous marker. This study suggests a potential molecular mechanism for how blood flow mediates DA and CV formation, whereby flow promotes arterial and represses venous fate specification, which then ensures the proper separation of the two vessels during development.

## Results

### Abnormal fusions between the DA and CV in *Ncx1*^−/−^ mutant embryos lacking blood flow

Although previous studies have shown a role for blood flow in formation of the mouse embryonic vasculature, including in the DA^6,7^, the precise role of flow in the morphogenesis of the DA and the CV is still not completely understood. To gain further insight into this process, we examined DA and CV development in embryos that lack blood flow, using the *Ncx1* ^−/−^ mutant.

**Figure 1:**
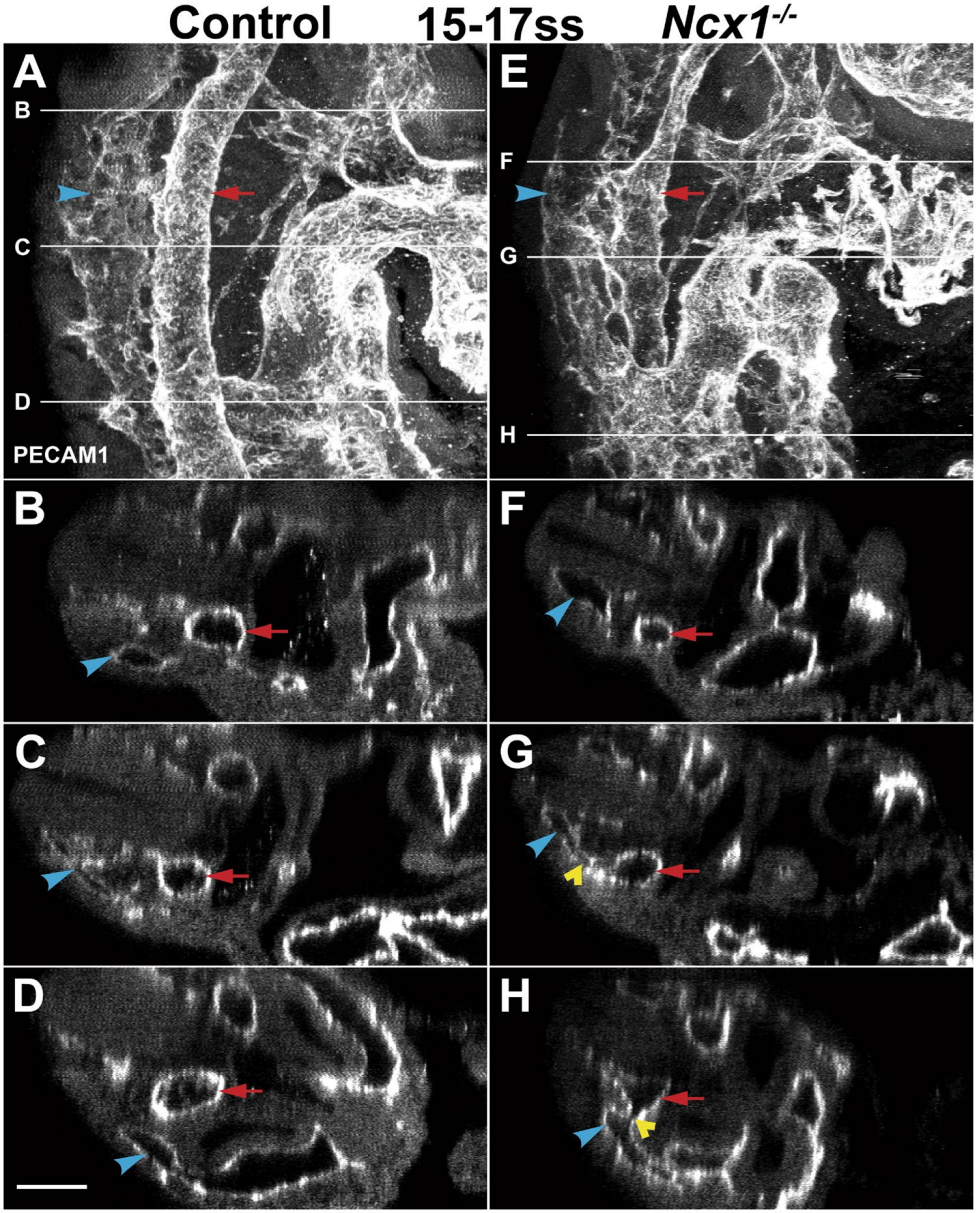
Abnormal fusions between the Dorsal Aorta (DA) and Cardinal Vein (CV) in *Ncx1* ^−/−^ mutants. Two-photon images of Control (**A**–**D**) and *Ncx1* ^−/−^ mutant (**E**–**H**) embryos at 15–17ss (E9.0) stained with PECAM-1. (**A**,**E**) Lateral projection view of the DA and CV. (**B**–**D**,**F**–**H**) Optical cross sections taken at indicated positions. Red arrow, DA; Blue arrowhead, CV; Yellow arrowhead, DA-CV fusion. Scale bar: 100 um. n = 3 embryos per genotype were analyzed.

To examine DA and CV morphogenesis in the *Ncx1* ^−/−^ mutant, we imaged the DA and CV in whole mount embryos by two-photon microscopy, which allowed us to reconstruct vessel morphology in detail. At 15–17ss (E9.0), in lateral views of the embryo in the region adjacent to the heart (Fig. 1A,E), or in optical cross sections through the embryo (Fig. 1B–D,F–H), the DA and CV in control mice were well separated vessels with distinct lumens. In all three *Ncx1* ^−/−^ mutants analyzed, there were abnormal fusions, where the two vessels appeared to be merged together (Fig. 1G,H, yellow arrowhead; average of 8.3 fusions per embryo). Furthermore, the DA in the *Ncx1* ^−/−^ mutant also appeared to be irregular, with the vessel larger at locations where fusions occurred and smaller in others, which suggested that DA irregularity may be related to the abnormal fusions. These results demonstrate that proper formation of the DA and CV was compromised, and abnormal fusions occurred between the two vessels in *Ncx1* ^−/−^ mutant embryos lacking blood flow.

### Abnormal fusions between the DA and CV were present shortly after the normal onset of blood flow

**Figure 2:**
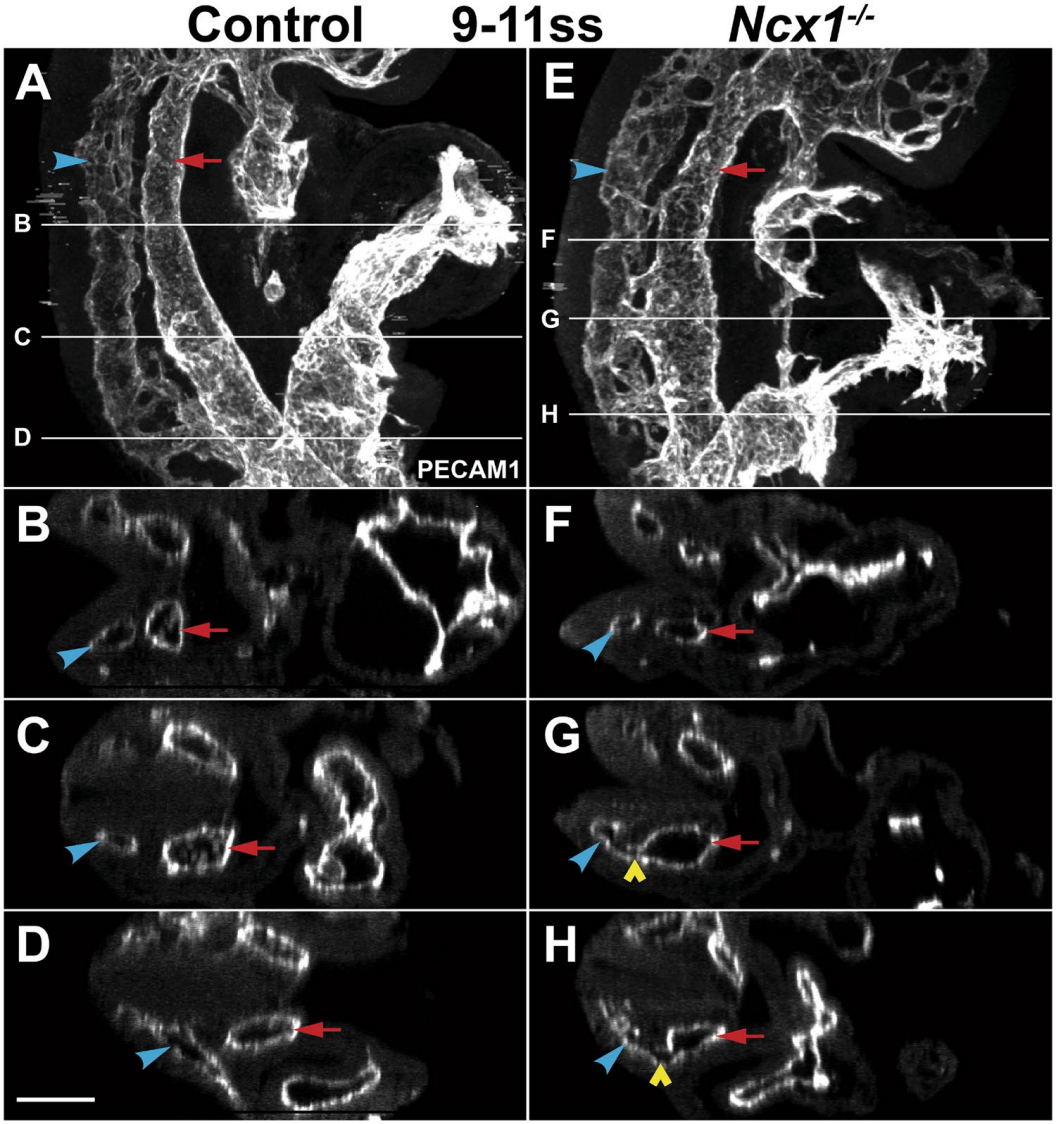
DA and CV fusions in the *Ncx1* ^−/−^ mutant were present at an embryonic stage shortly after the normal onset of blood flow. Two-photon images of Control (**A**–**D**) and *Ncx1* ^−/−^ mutant (**E**–**H**) embryos at 9–11ss (E8.0–8.5) stained with PECAM-1. Red arrow, DA; Blue arrowhead, CV; Yellow arrowhead, DA-CV fusion. Scale bar: 100 um. n ≥ 8 embryos per genotype were analyzed.

If the DA and CV fusions found in *Ncx1* ^−/−^ mutants were a direct result of the lack of blood flow, we would expect that fusions would be apparent soon after the stage when flow normally begins, which is around 6–7ss^4^. Therefore, we next examined the DA and CV shortly thereafter, at 9–11ss (E8.0–8.5). At this stage, very thin connections between the DA and the CV can be observed in control embryos, as previously reported^25^. However, 10 out of 16 *Ncx1* ^−/−^ mutants analyzed at this stage already displayed abnormal DA and CV fusions (Fig. 2E–H, yellow arrowhead; average of 3.5 fusions per embryo), which were not found in control embryos (Fig. 2A–D). Irregularity in the DA was also observed at this stage in the mutants (Fig. 2E,G, note the wider DA in the region where fusion is located). Therefore, the presence of the DA and CV defects and the initiation of blood flow in the embryo appeared to be correlated, supporting the hypothesis that flow is required to prevent improper fusions between the two vessels.

### Endothelial cells of the *Ncx1*^−/−^ mutant DA lacked expression of arterial markers

**Figure 3:**
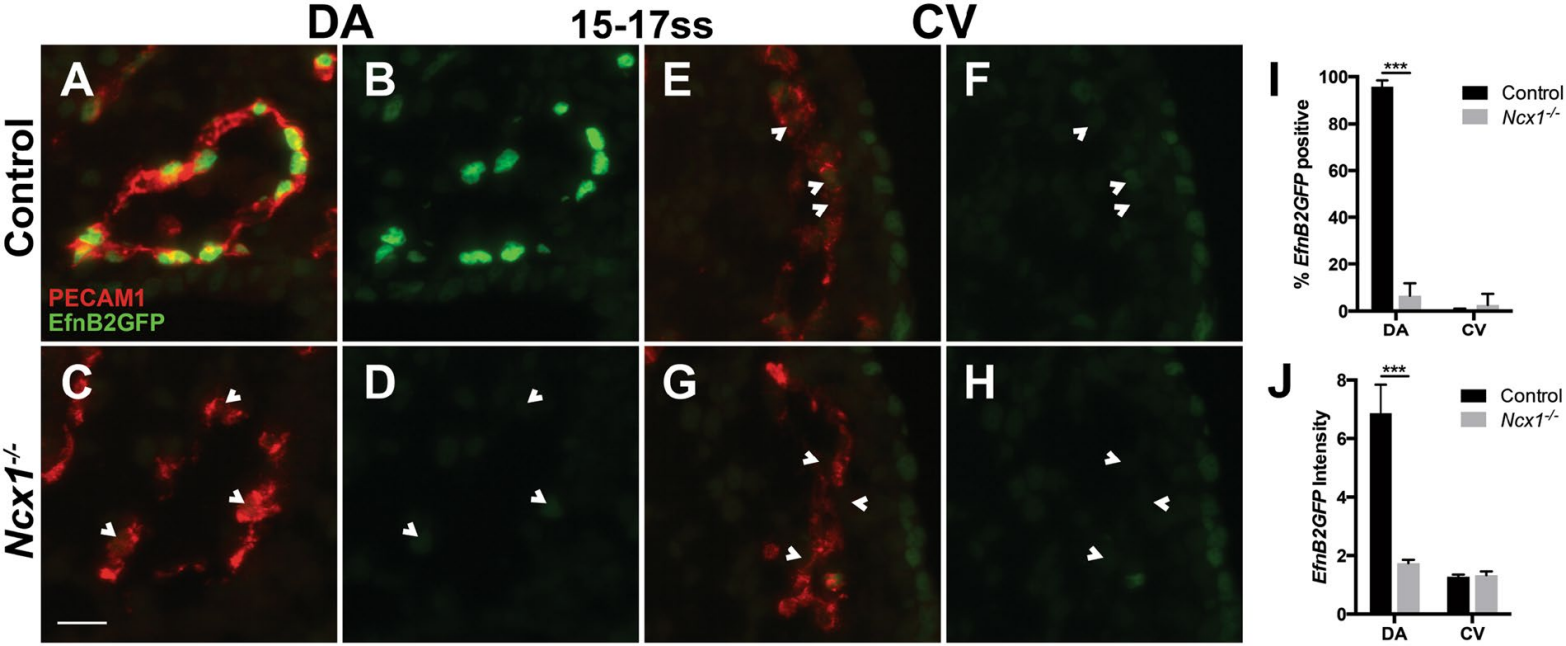
DA ECs lacked expression of the arterial marker ephrin-B2 in *Ncx1* ^−/−^ mutants. (**A**–**H**) EfnB2GFP expression at 15–17ss in ECs of control (**A**,**B**,**E**,**F**) and *Ncx1* ^−/−^ mutants (**C**,**D**,**G**,**H**) in the DA (**A**–**D**) and CV (**E**–**H**). Red, PECAM-1; Green, EfnB2GFP. Scale bar: 25 um. (**I**) Proportion of EfnB2GFP positive ECs in the DA and CV. (**J**) Average EfnB2GFP intensity per EC. ***P < 0.001. Average values  ±  s.d. are shown; n = 4 embryos per genotype.

**Figure 4:**
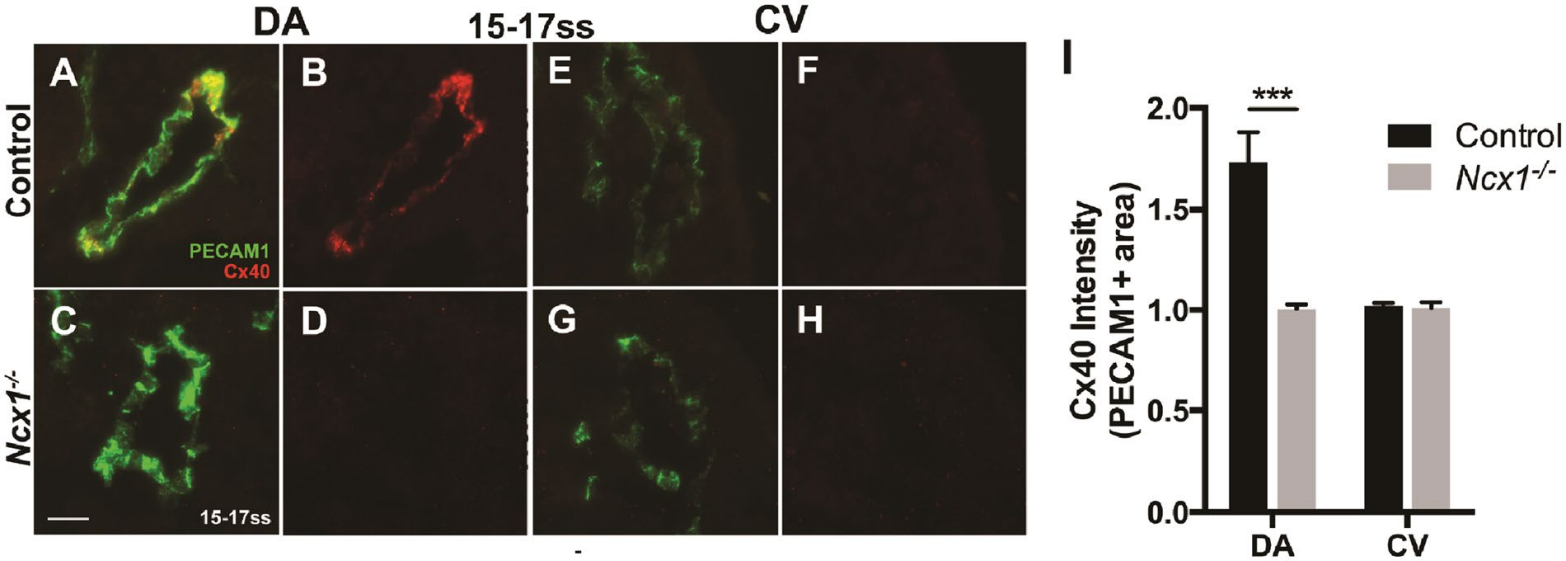
DA ECs in *Ncx1* ^−/−^ mutants also lacked expression of a second arterial marker, Connexin-40. (**A**–**H**) Cx40 expression at 15–17ss in ECs of control (**A**–**B**,**E**–**F**) and *Ncx1* ^−/−^ mutants (**C**,**D**,**G**,**H**) in the DA (**A**–**D**) and CV (**E**–**H**). Green, PECAM-1; Red, Cx40. Scale bar: 25 um. (**I**) Average Cx40 intensity (in PECAM1 positive area) in the DA and CV. ***P < 0.001. Average values  ±  s.d. are shown; n ≥ 4 embryos per genotype.

The aberrant fusions between the DA and CV in *Ncx1* ^−/−^ mutants suggested that their ECs were not restricted to the appropriate vessel. To test whether one underlying cause was a loss of proper arterial-venous identity in DA and CV ECs, we first examined arterial fate specification in the DA by looking at expression of the canonical arterial fate marker ephrin-B2, using the *EfnB2* ^*H2BGFP*^ reporter line, which contains a nuclear GFP (H2BGFP) transgene knocked into the *EfnB2* gene locus^26^. At 15–17ss, in control embryos, nearly all ECs of the DA were *EfnB2GFP* positive (96 ± 3%, Fig. 3A,B,I), whereas in the *Ncx1* ^−/−^ mutant, very few DA ECs were (7 ± 5%, p < 0.001, Fig. 3C,D,I). The average level of *EfnB2GFP* per cell in the DA was also much decreased in the *Ncx1* ^−/−^ mutant compared to controls (1.7 ± 0.1 vs. 6.9 ± 1.0, p < 0.001, Fig. 3J). ephrin-B2 is known to be expressed in the DA but not the CV^18^, and *EfnB2GFP* expression was not observed in the CV of control or mutant embryos (Fig. 3E–H,I,J). To confirm that DA ECs in the *Ncx1* ^−/−^ mutant lacked proper arterial specification, we looked at a second arterial marker, the gap junction protein Connexin-40 (Cx40). In control embryos, Cx40 was expressed in the DA but not the CV (Fig. 4A,B,E,F,I), as was shown previously^1,20^. Consistent with the findings from examining *EfnB2GFP* expression, DA expression of Cx40 was also lost in *Ncx1* ^−/−^ mutants (Fig. 4C,D,G–I). Therefore, these results suggested that the DA and CV fusions seen in *Ncx1* ^−/−^ mutants were accompanied by a lack of arterial specification in ECs of the DA.

### Increased arterial marker expression in the DA following the onset of blood flow was lost in *Ncx1*^−/−^ mutants

We sought to examine in more detail the requirement of flow in arterial fate specification in DA ECs. If the onset of flow acts as a signal that contributes to the acquisition of arterial fate in ECs in the DA, we might expect arterial marker expression in DA ECs to increase following the onset of blood flow, in a flow-dependent manner.

**Figure 5:**
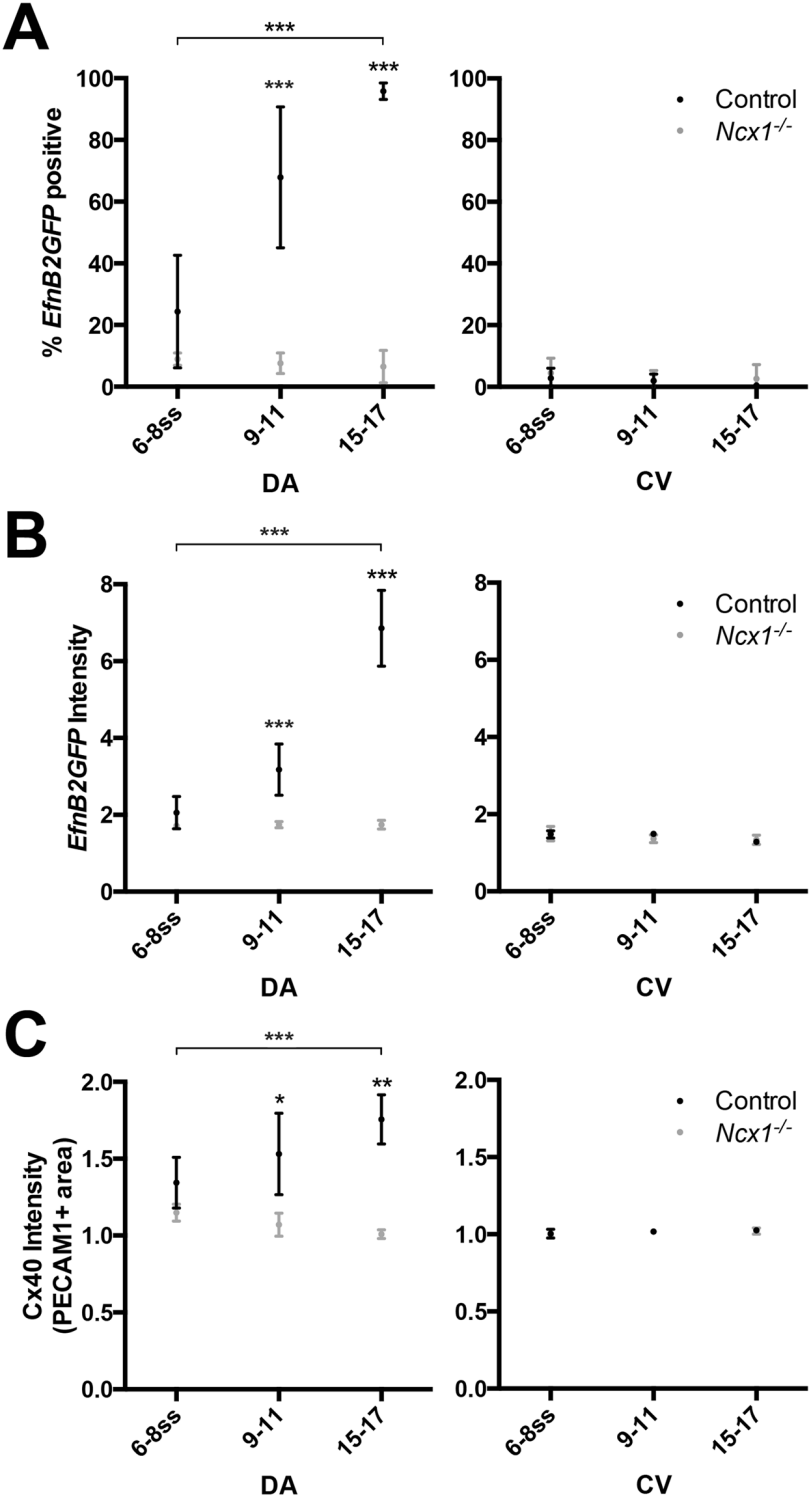
Arterial marker expression in DA ECs increased at somite stages following the onset of blood flow, which was lost in the *Ncx1* ^−/−^ mutant. (**A**) Proportion of *EfnB2GFP* positive ECs at 6–8, 9–11, and 15–17ss in controls and *Ncx1* ^−/−^ mutants, in the DA and the CV. (**B**) Average *EfnB2GFP* intensity per EC in the DA and CV. (**C**) Average Cx40 intensity (in PECAM1 positive area) in the DA and CV. Statistical analysis was performed using an ANOVA with Bonferroni post test. *P < 0.05, **P < 0.01, ***P < 0.001. Average values  ±  s.d. are shown; n ≥ 3 embryos per genotype.

To test this hypothesis, we analyzed EC expression of *EfnB2GFP* and Cx40 over time, beginning at the stage when blood flow is initiated (6–8ss), in control and *Ncx1* ^−/−^ mutant embryos. At this stage, *EfnB2GFP* expression was found in 27 ± 18% of DA ECs in control embryos. By 9–11ss, expression increased to 68 ± 23% of DA ECs, and by 15–17ss, nearly all DA ECs were *EfnB2GFP* positive (96 ± 3%, Fig. 5A). Average *EfnB2GFP* intensity per cell also increased over this time period (Fig. 5B), which, together with the previous findings, suggested that *EfnB2GFP* expression in DA ECs was correlated with the initiation of blood flow. Furthermore, this acquisition of *EfnB2GFP* expression was flow-dependent, as it was abrogated in the *Ncx1* ^−/−^ mutant (Fig. 5A,B). These data, therefore, suggested that DA ECs acquired *EfnB2GFP* expression following the initiation of blood flow, and required flow to do so.

We found a similar result upon examining Cx40 expression in DA ECs. Cx40 intensity in the DA increased from 6–8ss to 15–17ss (1.35 ± 0.16 to 1.73 ± 0.15, p < 0.05), and this increase failed to occur in the *Ncx1* ^−/−^ mutant (Fig. 5C). However, the expression of the arterial marker Dll4 was not affected in mutant embryos (data not shown), consistent with previously published data^1^. Altogether, these results suggested that in the DA, arterial marker expression was correlated with the onset of blood flow, in a flow-dependent manner, suggesting a role for flow in acquisition of arterial fates of DA ECs, even if this was restricted to a subset of arterial markers.

### Reduced Notch1 activity in endothelial cells of the DA in *Ncx1*^−/−^ mutants

**Figure 6:**
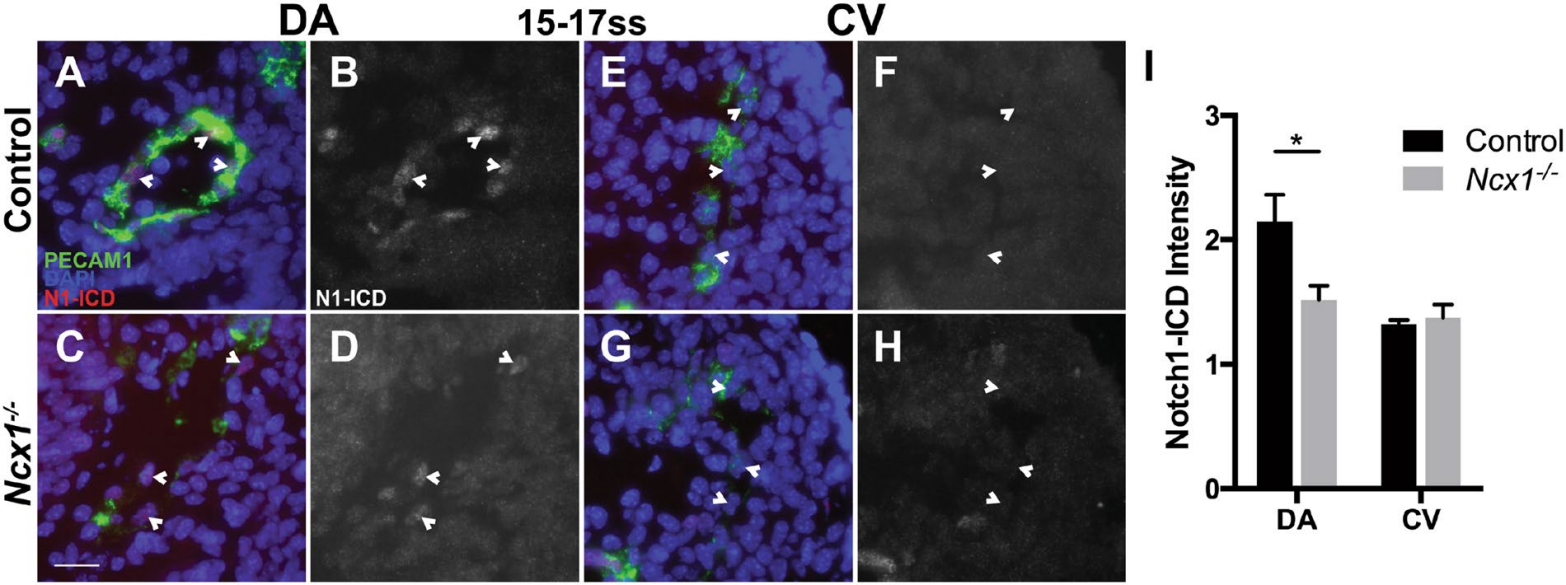
Reduced Notch1 activation in DA ECs in *Ncx1* ^−/−^ mutants. (**A**–**H**) Notch1-ICD (N1-ICD) expression at 15–17ss in ECs of controls (**A**,**B**,**E**,**F**) and *Ncx1* ^−/−^ mutants (**C**,**D**,**G**,**H**) in the DA (**A**–**D**) and CV (**E**-**H**). Green, PECAM-1; Blue, DAPI; Red, Gray, N1-ICD. Scale bar: 25 um. (**I**) Average Notch1-ICD intensity per EC in the DA and CV. *P < 0.05. Average values  ±  s.d. are shown; n = 3 embryos per genotype.

To investigate the molecular mechanism underlying the requirement of blood flow in arterial fate specification in the DA, we next examined the Notch pathway, which promotes arterial fates in endothelial cells and is required for arterial marker expression in the DA^17,23^. To determine whether Notch signaling may also be affected in *Ncx1* ^−/−^ mutants, we assayed Notch activity in the DA using an antibody against the cleaved, activated form of Notch1 (Notch1-ICD). Notch1-ICD staining was found in the DA in control embryos (Fig. 6A,B), but in *Ncx1* ^−/−^ mutants, Notch1-ICD intensity was significantly reduced in the DA (Fig. 6C,D,I, 2.2 ± 0.2 vs. 1.5 ± 0.1, p < 0.05). As expected, Notch1 signaling did not appear to be activated in the CV (Fig. 6E-I). This is consistent with a previous study showing that Notch1 expression requires flow during development^24^.Therefore, our findings indicated that Notch1 was also activated in the DA in a flow-dependent manner, suggesting one potential molecular mechanism for the requirement of flow in arterial fate specification.

### Expression of the venous marker Coup-TFII was mildly affected in endothelial cells of the CV in *Ncx1*^−/−^ mutants

**Figure 7:**
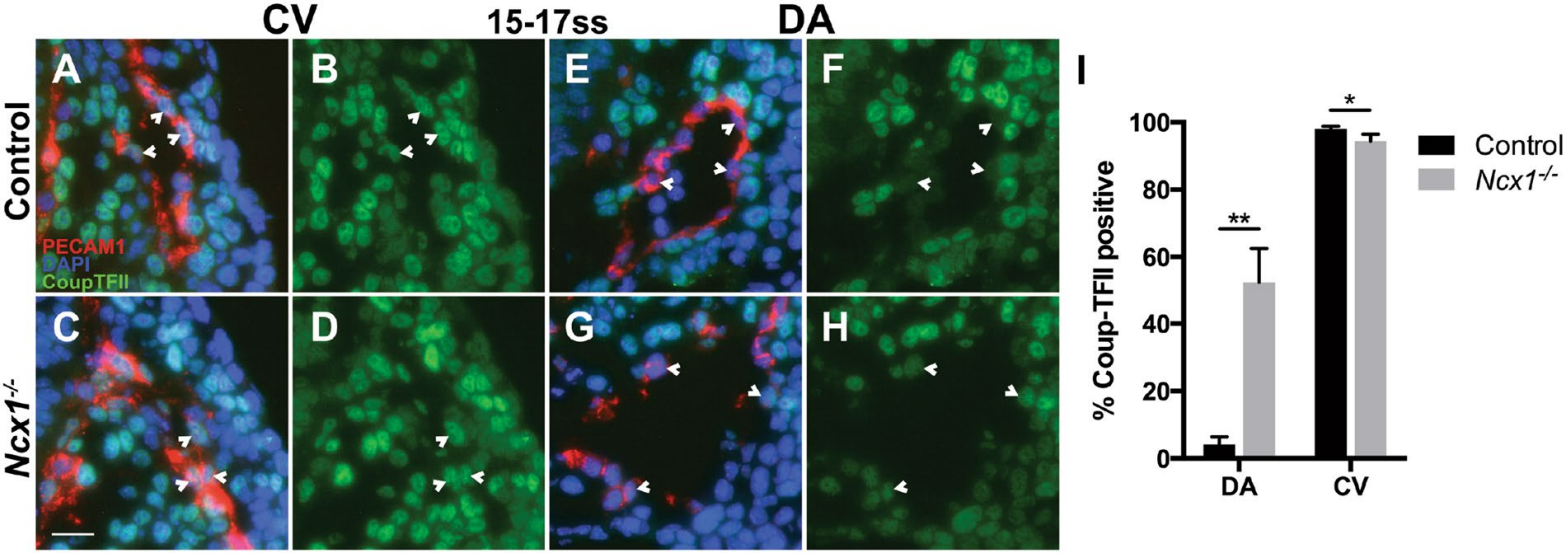
Expression of the venous marker Coup-TFII in *Ncx1* ^−/−^ mutants. (**A**–**H**) Coup-TFII expression at 15–17ss in ECs of controls (**A**,**B**,**E**,**F**) and *Ncx1* ^−/−^ mutants (**C**,**D**,**G**,**H**) in the CV (**A**–**D**) and DA (**E**–**H**). Red, PECAM-1; Blue, DAPI; Green, Coup-TFII. Scale bar: 25 um. (**I**) Proportion of Coup-TFII positive ECs in the DA and CV. *P < 0.05, **P < 0.01. Average values  ±  s.d. are shown; n = 4 embryos per genotype.

The results of our previous experiments showed that the DA and CV fusions in *Ncx1* ^−/−^ mutants were accompanied by loss of arterial fates in the DA, which supports the hypothesis that the fusions may result from the loss of proper arterial-venous identity that distinguishes DA and CV ECs. We next tested whether venous fate of CV ECs was similarly affected in *Ncx1* ^−/−^ mutants by examining expression of the venous marker Coup-TFII^27^. In control embryos, Coup-TFII was expressed in the CV (Fig. 7A,B). In *Ncx1* ^−/−^ mutants, the proportion of Coup-TFII expressing cells was slightly reduced, although most CV ECs retained Coup-TFII expression (98 ± 1% vs. 94 ± 2%, p < 0.05, Fig. 7C,D,I). Therefore, in contrast to arterial fate specification in the DA, venous fate specification in the CV was less affected in *Ncx1* ^−/−^ mutants lacking blood flow.

### Ectopic expression of the venous marker Coup-TFII in endothelial cells of the DA in *Ncx1*^−/−^ mutants

Although Coup-TFII expression was affected mildly in *Ncx1* ^−/−^ mutants in the CV, in the DA we were surprised to find ectopic Coup-TFII expression. In 15–17ss control embryos, only rare Coup-TFII positive DA ECs were found (4 ± 2%, Fig. 7E,F,I), as has been reported previously^20^. In *Ncx1* ^−/−^ mutants however, we found that approximately half of DA ECs expressed Coup-TFII (53 ± 10%, Fig. 7G,H,I), suggesting that flow was required to suppress ectopic expression of this venous marker in the DA. Therefore, our findings suggest a requirement for blood flow in DA EC fate specification, both for promoting arterial marker and for suppressing venous marker expression.

### No significant changes in the number of endothelial cells in the DA and CV of *Ncx1*^−/−^ mutants

**Figure 8:**
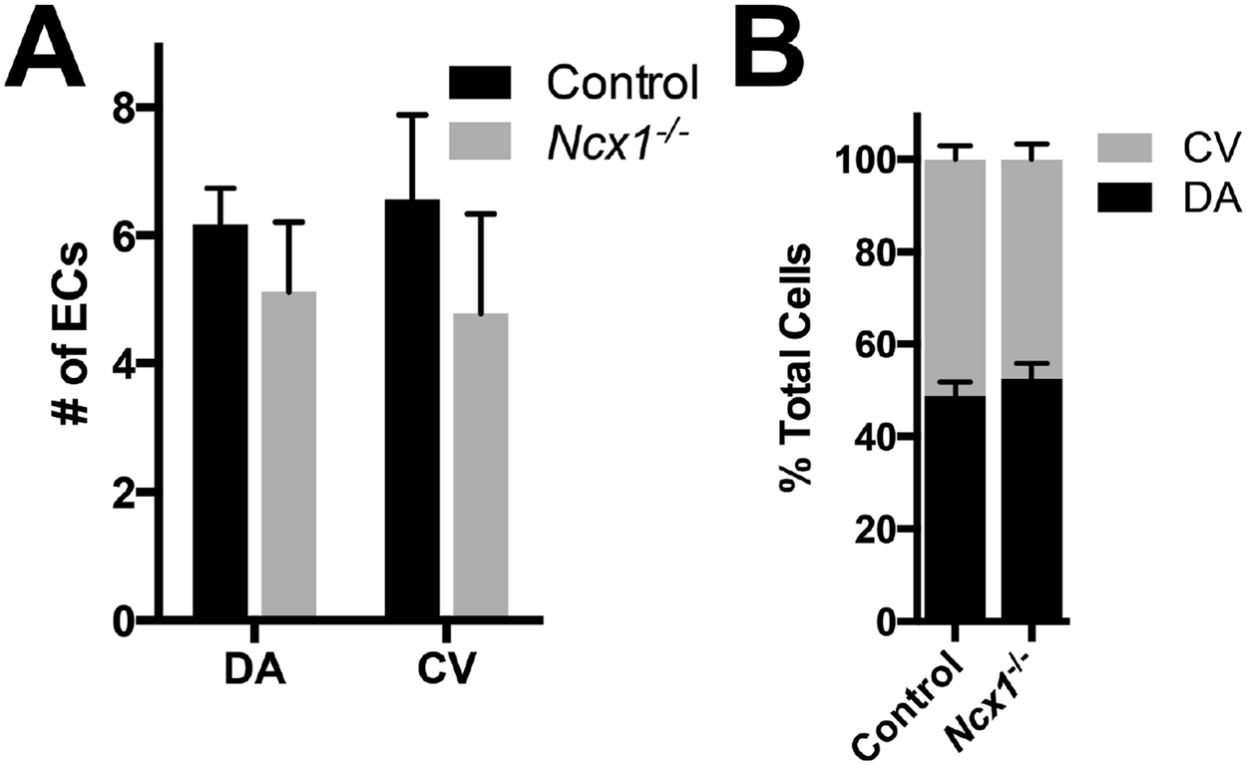
No significant differences were found in the number or distribution of ECs in *Ncx1* ^−/−^ mutants. (**A**) Average number of ECs per vessel section in the DA and CV. (**B**) Distribution of ECs between the DA and CV. Average values  ±  s.d. are shown; n = 4 embryos per genotype.

Our previous experiment showed that in *Ncx1* ^−/−^ mutants approximately half of DA ECs were Coup-TFII positive, whereas only rare Coup-TFII positive ECs were present in the DA in controls. To confirm that this was due to an increase in ectopic Coup-TFII expression in DA ECs, rather than redistribution of ECs from the CV, we counted the number of ECs in both the DA and the CV, in controls and *Ncx1* ^−/−^ mutants. In the DA, we found an average of 6.2 ± 0.6 ECs per vessel section in controls and 5.1 ± 1.1 ECs in *Ncx1* ^−/−^ mutants, a difference which was not statistically significant (Fig. 8A, p = 0.15). In the CV there were 6.6 ± 1.3 vs. 4.8 ± 1.6 ECs in controls and *Ncx1* ^−/−^ mutants, respectively, which was also not a significant difference (Fig. 8A, p = 0.13). Importantly, the relative distribution of ECs between the DA and CV also did not change in controls versus *Ncx1* ^−/−^ mutants (Fig. 8B). As the arteriovenous expression pattern of the CV in *Ncx1* ^−/−^ mutants was only slightly affected, these data together suggest that there was no redistribution of ECs from the CV to the DA. These results therefore support the hypothesis that the Coup-TFII positive cells found in the DA indicate ectopic Coup-TFII expression by DA ECs.

## Discussion

Our study of the development of the mouse embryonic DA and CV in *Ncx1* ^−/−^ mutants lacking a heartbeat, and thus blood flow, reveals an important role for flow in the morphogenesis of this parallel artery and vein pair. Our results suggest that flow mediates proper formation of the DA and CV into two separate vessels and prevents the development of abnormal fusions between them. Our experiments provide *in vivo* evidence that flow is essential for proper arterial-venous molecular specification of the ECs of the DA, by promoting the acquisition of arterial molecular markers and suppressing improper expression of venous markers. In contrast to its role in the DA, flow is less important in arterial-venous fate specification of ECs in the CV. We therefore propose a model whereby blood flow induces arterial while suppressing venous molecular programming in the DA. In the absence of flow, the expression of arterial markers was lost, whereas expression of venous markers was increased in DA ECs. This abnormal arterial-venous marker expression pattern more closely resembles the pattern in CV ECs. These molecular defects may contribute to the failure of proper DA and CV separation and the presence of fusions between the two vessels.

Imaging the vasculature in whole-mount embryos by two-photon imaging allowed for a high-resolution three-dimensional reconstruction of the DA and CV and detailed examination of their morphology and structure. Using this method, we could investigate the role of blood flow on DA and CV formation by examining embryos mutant for *Ncx1*, which leads to a lack of heartbeat and thus blood flow without affecting the cells of the vasculature directly. These analyses suggest that flow is critically required to ensure that the DA and CV develop as two separate vessels, and do not develop abnormal fusions between them. Our finding proposes for the first time that blood flow is required for proper formation of paired artery and vein during mammalian vascular morphogenesis.

Our work also suggests that flow is required for the development of the normal caliber of the DA. We found that the *Ncx1* ^−/−^ mutant DA had an irregular diameter, with certain regions wider and other regions narrower throughout the length of the vessel. This finding is consistent with that from a previous study on a mutant in the striated muscle protein *titin*, which has defects in heartbeat and delayed blood flow that also leads to an irregular DA^6^. Together, the results from both studies suggest that the irregular DA phenotype is indeed a consequence of the lack of flow. Our current study further shows that the regions with a wider DA are regions of DA and CV fusion. Thus, our work shows for the first time that flow is essential for the development of both the DA and CV, preventing fusions between the two vessels.

Our study uncovers a crucial role of blood flow in the proper arterial specification of DA ECs. In the absence of blood flow, the DA did not exhibit expression of the arterial markers *EfnB2GFP* and Cx40. Consistent with this result, expression of Cx40 in the DA has also been shown to be abolished when blood flow was physically ablated *ex vivo* and embryos were subsequently grown in culture^1^. Furthermore, we found that expression of these arterial markers increased in somite stages following the onset of blood flow, in a flow-dependent manner, suggesting that flow may act as a signal that contributes to the acquisition of arterial fate by DA ECs in early development.

Notch signaling plays an important role in arterial fate specification, including in ECs of the DA^17,19,23^. In *Ncx1* ^−/−^ mutants, we found that Notch1 activation decreased in DA ECs. This finding is consistent with an earlier study showing loss of *Notch1* mRNA in the DA in embryos where blood flow was physically ablated^24^. Our finding on Notch1 activation in developing embryos in utero, together with that of Notch1 mRNA in embryos cultured *ex vivo*, lead us to conclude that blood flow is required to increase Notch signaling in the DA to promote arterial fates.

In addition to its role in promoting arterial fate in the DA, our analysis of *Ncx1* ^−/−^ mutants also shows for the first time that blood flow suppresses the expression of the venous marker Coup-TFII in DA ECs. Previous studies of embryos lacking blood flow in the mouse have not reported the expression of venous fate markers. However, in the yolk sac of the chick, ligation of the vitelline artery and subsequent blockade of arterial flow leads to ectopic expression of a number of venous markers, including Coup-TFII, along with downregulation of arterial markers^28,29^. Therefore, we believe that blood flow plays a role in promoting arterial and suppressing venous fate specification in DA ECs.

Blood flow is crucially important for arterial-venous fate specification in DA ECs, but appears to play less of a role in the CV. In the absence of flow, arterial markers, including Notch1, ephrin-B2, and Cx40 in CV ECs remain unexpressed, whereas expression of the venous marker Coup-TFII is mildly affected. This suggests that blood flow likely affects arterial-venous specification in CV ECs to a lesser extent than it does in DA ECs. One possible cause for this difference is that flow rates and hemodynamic forces in the CV may be lower than those found in the DA. Indeed, such a difference has been measured at this stage in the developing yolk sac, where flow rates in veins were found to be >2x less than those in arteries^30^.

The precise mechanism by which blood flow ensures proper separation of the DA and CV is not completely understood. Our study suggests a potential mechanism, whereby blood flow promotes arterial-venous specification of DA ECs, which then ensures DA and CV separation during development. In the absence of flow, DA ECs lose arterial and gain ectopic venous marker expression, giving them a molecular profile more similar to that of CV ECs. This lack of molecular differentiation between DA and CV ECs may ultimately be responsible for their failure to properly separate or remain separated during development, leading to fusions between the two vessels. In support of this notion, a previous study has examined mutants in *Notch1* and *EfnB2*, two of the genes which showed reduced or absent expression in the DA in the absence of blood flow. Although these mutants had several defects, including changes in the size of the DA, they also contained arterial-venous shunts between the DA and the venous circulation, supporting the idea that loss of arterial DA EC fate can lead to fusions between the two vessels^21^.

EC polarity and directed cell movement have also been shown to play important roles during blood vessel formation, as the direction of EC movement within a vessel is sensitive to the rate and direction of flow. By using a computational model to calculate blood flow and shear stress forces in the vasculature of the developing postnatal retina^31^, others have shown showed that ECs are polarized against the simulated direction of flow, and that the strength of polarization is correlated with vessel-wall shear-stress levels. Another recent study using live imaging of the yolk sac vasculature found that ECs in arterial vessels migrate towards regions of greater vessel diameter against the direction of flow, as well as from vessels experiencing low blood flow velocities into those with high flow velocities^30^. It is therefore possible that in the absence of blood flow in the *Ncx1* ^−/−^ mutant, aberrant EC polarity and EC movement may underlie the abnormal fusions between the DA and CV. Together, blood flow and proper arterial-venous EC specification may affect polarity and directed cell movement, which are crucial for the morphogenesis of the developing DA and CV.

## Materials and Methods

### Mice

*Ncx1* ^*lacZ*^ mice^11^, kindly provided by Dr. Simon J. Conway, and *EfnB2-H2BGFP* mice^26^, have been described previously. Mice used in this study were kept in a mixed genetic background. All experimental protocols were approved by the University of California, San Francisco Institutional Animal Care and Use Committee, and all experimental methods were carried out in accordance with the approved protocols.

### Immunofluorescence staining

Embryo harvest, processing, and immunostaining were performed as described^20,32^. Briefly, for whole-mount staining, embryos were dissected and fixed with 4% paraformaldehyde overnight at 4°, washed in PBS, and incubated in blocking solution (5% donkey serum and 0.1% Triton X-100 in PBS). After blocking, embryos were incubated in primary antibodies, washed, and incubated in secondary antibodies. All incubations were carried out for 12 h at 4° with rocking. After serial dehydration in 25, 50, 75, and 100% ethanol at 30-minute intervals, embryos were then cleared in methylsalicylate before imaging.

For frozen sections, embryos were fixed in 4% paraformaldehyde for 20 minutes on ice, embedded in OCT, and sectioned. Sections were blocked in 5% donkey serum and 0.2% Triton X-100 in PBS for 1 hour at room temperature. They were then incubated with primary antibody in blocking solution overnight at 4°, washed, incubated in secondary antibody for 1 hour at room temperature, and mounted in DAPI-containing Vectashield (Vector Laboratories).

The following primary antibodies were used: rat anti-CD31 (1:200; 550274, BD Biosciences), goat anti-Connexin-40 (1:2000; sc-20466, Santa Cruz Biotechnology), mouse anti-Coup-TFII (1:100; PP-H7147–00, R&D Systems). Secondary antibodies were: donkey anti-rat IgG Alexa488 (Invitrogen), donkey anti-goat IgG Alexa555 (Invitrogen), donkey anti-rat IgG Cy3 (Jackson Immunoresearch), and donkey anti-mouse IgG Cy5 (Jackson Immunoresearch). All secondary antibodies were used at 1:500.

Images were acquired on a Zeiss Axiovert2 Plus microscope or custom-built two-photon microscope (see below) and analyzed with ImageJ and Imaris (Bitplane).

### Two-photon microscopy and image analysis

Images were acquired using a locally constructed two-photon microscope^33^. Whole-mount embryos were imaged by acquiring stacks of planar images with 1 μm spacing along the optical axis. The data were reconstructed in three dimensions and analyzed with Imaris software (Bitplane Scientific Software).

### Quantification of antibody staining and expression

Sections (10 μm) taken from the region of the embryo from the top of aortic arch to the region where the CV merges with the common cardinal vein were analyzed. To quantify staining intensities, captured images were exported as 16-bit TIFF files and analysis was performed in ImageJ. For *EfnB2GFP-H2BGFP*, DAPI-positive nuclei in CD31-labeled cells of the DA or CV were identified and the corresponding staining intensity was determined. To control for variation between embryos, the intensity for each cell was normalized against the average value of the five lowest cell intensities in either the DA or the CV in the corresponding embryo. A normalized GFP intensity of 2.5 or higher was scored as positive. For Cx40, the PECAM1 positive region of the DA or CV was determined by thresholding, and average staining intensity in this region was measured and normalized against tissue background. For Coup-TFII, cells were scored as positive or negative by visual inspection.

### Statistical analysis

Values are expressed as mean ± s.d. The two-tailed Student’s *t*-test was used for comparisons between two groups except for Fig. 5 when an ANOVA test was used and specified in the figure legend. *P* < 0.05 was considered significant.
